# Effects of Magnesium, Pyrophosphate and Phosphonates on Pyrophosphorolytic Reaction of UDP-Glucose Pyrophosphorylase

**DOI:** 10.3390/plants11121611

**Published:** 2022-06-20

**Authors:** Leszek A. Kleczkowski, Daniel Decker

**Affiliations:** Umeå Plant Science Center, Department of Plant Physiology, Umeå University, 90187 Umeå, Sweden; danielodecker86@gmail.com

**Keywords:** chemical genetics, Dixon plot, fosetyl-Al, inhibitor kinetics, magnesium activation, *Phytophthora*

## Abstract

UDP-glucose pyrophosphorylase (UGPase) carries a freely reversible reaction, using glucose-1-P and UTP to produce UDP-glucose (UDPG) and pyrophosphate (PP_i_), with UDPG being essential for glycosylation reactions in all organisms including, e.g., synthesis of sucrose, cellulose and glycoproteins. In the present study, we found that free magnesium (Mg^2+^) had profound effects on the reverse reaction of purified barley UGPase, and was absolutely required for its activity, with an apparent *K*_m_ of 0.13 mM. More detailed analyses with varied concentrations of MgPP_i_ allowed us to conclude that it is the MgPP_i_ complex which serves as true substrate for UGPase in its reverse reaction, with an apparent *K*_m_ of 0.06 mM. Free PP_i_ was an inhibitor in this reaction. Given the key role of PPi in the UGPase reaction, we have also tested possible effects of phosphonates, which are analogs of PPi and phosphate (P_i_). Clodronate and etidronate (PP_i_ analogs) had little or no effect on UGPase activity, whereas fosetyl-Al (P_i_ analog), a known fungicide, acted as effective near-competitive inhibitor versus PP_i_, with *K*_i_ of 0.15 mM. The data are discussed with respect to the role of magnesium in the UGPase reaction and elucidating the use of inhibitors in studies on cellular function of UGPase and related enzymes.

## 1. Introduction

UDP-glucose (UDPG) pyrophosphorylase (UGPase) is a freely reversible enzyme that uses glucose-1-P (Glc-1-P) and uridine-triphosphate (UTP) in its forward (synthesis) reaction and UDPG and inorganic pyrophosphate (PP_i_) in the reverse direction (pyrophosphorolysis) [1,2]. Both the synthesis and pyrophosphorolysis reactions of UGPase are deemed essential for plants, with the former providing UDPG for hundreds of glycosylation reactions, and the pyrophosphorolysis reaction involved in energy production (UTP) [3,4] and providing carbon skeletons in the sucrose-to-starch pathway in certain non-photosynthetic tissues [5,6,7]. For both directions of the reaction, it has been reported that magnesium is required for activity [1,8,9,10]. For the pyrophosphorolysis reaction, it has been suggested that this magnesium requirement is consistent with the chelation of PP_i_ to form MgPP_i_ [1,9,11,12], which may represent the true substrate of the reaction. However, no kinetic studies with defined concentrations of MgPP_i_ versus UGPase activity have been carried out, leaving out the possibility, for instance, that magnesium may act independently of PP_i_, serving as a cofactor rather than as a part of the MgPP_i_ complex.

It has been estimated that about 85% of total adenosine-triphosphate (ATP), ca. 40% of total adenosine-diphosphate (ADP), and 5% of total adenosine-monophosphate (AMP) are complexed with magnesium under conditions of 0.5 mM free magnesium (Mg^2+^) [13,14], i.e., conditions characteristic of plant cell cytosol, where UGPase activity is located. The same degree of Mg-complexation occurs also for other nucleotides (e.g., uridylates) [7]. Other phosphorylated compounds (e.g., PP_i_, UDPG or Glc-1-P) can also bind Mg^2+^, depending on their stability constants with this metal [15]. As intracellular [Mg^2+^] undergoes fluctuations responding to changes in adenylate status in a given compartment and due to activities of Mg^2+^-translocators, the concentrations of specific Mg-complexes may also change accordingly [14,16]. This may be especially important under anoxic/hypoxic conditions, when production of ATP is inhibited and cytosolic [Mg^2+^] is elevated, increasing its potential to chelate other phosphorylated metabolites [3].

In the present study, we used purified barley UGPase to test the effects of magnesium on activity of the enzyme and to test the possibility that MgPP_i_ is the true substrate in the pyrophosphorolysis reaction. A second goal of these studies was to examine effects of phosphonates as potential inhibitors of UGPase reaction. Phosphonates are structurally related to PP_i_ and are thus likely to affect PP_i_-dependent activities [17]. Phosphonates have also been known for long time as promising fungicides [18,19]. Overall, it has been found that MgPP_i_ rather than free PP_i_ serves as the true substrate of UGPase in the pyrophosphorolysis direction, with free PP_i_ inhibiting the reaction. As for the effect of phosphonates, two bisphosphonates (clodronate and etidronate) turned out to be largely inert, while fosetyl-Al (a monophosphonate) was an efficient near-competitive inhibitor of UGPase.

## 2. Results and Discussion

### 2.1. Is Magnesium Required as Substrate for UGPase?

In an earlier work it was shown that PP_i_ at high concentrations inhibits the pyrophosphorolytic reaction (from PP_i_ and UDPG) of barley UGPase, and that the inhibition can be partly relieved by increased [MgCl_2_] [20]. This observation has suggested that there is another factor (probably Mg^2+^) required for PP_i_ to form a complex with, that acts as true substrate. In some other PP_i_-utilizing enzymes, e.g., pyrophosphatases, it is MgPP_i_ rather than free PP_i_ that serves as substrate of the reaction [21,22].

When in a mixture, Mg^2+^ binds PP_i_ to form MgPP_i_, with the stability constant (*K*) of 1.2 mM^−1^ [23]. The reaction can be presented as:

Mg + PP_i_ <--> MgPP_i_

and can be mathematically described by the following equation:

*K* = *x*/[(*M* − *x*)(*A* − *x*)]

where *K* is stability constant for MgPP_i_, *M* is total [Mg], *A* is total [PP_i_], and *x* is [MgPP_i_]. Using that as a starting point, one can derive a simple quadratic equation:

*Kx*^2^ − *x*(*KM* + *KA* + 1) + *KMA* = 0

which, after conversions, can be presented as:

$$x=\frac{\left(KM+KA+1\right)\pm \sqrt{{\left(KM+KA+1\right)}^{2}-4K^{2}MA}}{2K}$$

Since *K*, *M* and *A* are known, once *x*, i.e., [MgPP_i_], is calculated, one can also calculate the concentration of Mg^2+^ as (*M* − *x*), and that of free PP_i_ as (*A* − *x*). Generally, at a physiological concentration of cytosolic Mg^2+^ of ca. 0.5–1 mM, [MgPP_i_] stabilizes at about 40–60% of [PPi_total_] [14].

**Figure 1:**
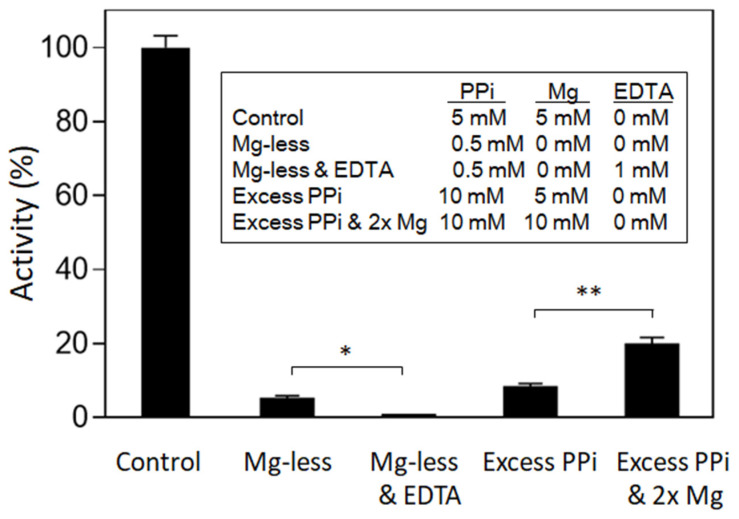
Effects of Mg^2+^ and PP_i_ on pyrophosphorolytic activity of barley UGPase. Standard reaction mixture assays (in triplicates) were used, with the exception of varying concentrations of PP_i_ and MgCl_2_ (Mg), as indicated. Statistical significance between the samples: * (*p* < 0.05); ** (*p* < 0.01).

In this study, the activity of purified barley UGPase was tested with and without MgCl_2_ (Figure 1). With no magnesium, the enzyme had low residual activity, which completely disappeared upon addition of ethylene diaminetetraacetic acid (EDTA), a strong chelator of divalent cations. This has strongly suggested that magnesium is essential for UGPase activity. PP_i_ at 10 mM clearly served as a strong inhibitor of UGPase at 5 mM MgCl_2_, whereas an increase in [MgCl_2_] to 10 mM partially relieved the PP_i_-dependent inhibition. Taking into account the stability constant for MgPP_i_ of 1.2 M^−1^ [23], under conditions of “PP_i_ excess” (total PP_i_ at 10 mM, MgCl_2_ at 5 mM), MgPP_i_ and free Mg (Mg^2+^) were at about 4.4 and 0.6 mM, respectively. At “PP_i_ excess and double MgCl_2_” (10 mM PP_i_ and 10 mM MgCl_2_), MgPP_i_ and Mg^2+^ were at 7.5 and 2.5 mM, respectively. These data have suggested that MgPP_i_ serves as true substrate for UGPase, whereas free PP_i_ is an inhibitor to the reaction.

**Figure 2:**
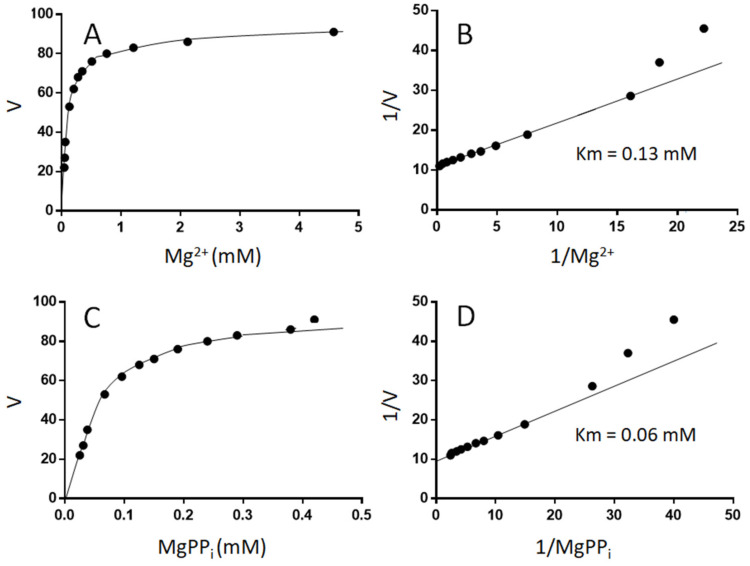
Effects of Mg^2+^ and MgPP_i_ on activity of barley UGPase in its pyrophosphorolytic reaction. (**A**) UGPase activity (V) versus [Mg^2+^]; (**B**) Double reciprocal plot for data in panel (**A**); (**C**) UGPase activity (V) versus [MgPP_i_]; (**D**) Double reciprocal plot for data in panel (**C**).

To probe in more detail the interactions between the enzyme, magnesium and PP_i_, the effects of magnesium on UGPase activity were studied kinetically with defined concentrations of Mg^2+^ and MgPP_i_. The activity of UGPase versus Mg^2+^ followed what appeared to be a typical hyperbolic curve (Figure 2A), consistent with Michaelis–Menten kinetics [24]. This has suggested that magnesium is true substrate of the reaction. However, it was not clear whether it was the result of Mg^2+^ binding directly to the enzyme (and serving as cofactor) or binding first to some other ligand(s) and serving as a substrate only in a complexed form (e.g., as MgPP_i_ and/or MgUDPG). When [MgPP_i_] was calculated for each of the experimental points and plotted versus observed activity, the resulting plot again appeared hyperbolic (Figure 2C). However, when these data were analyzed using double reciprocal plots for both Mg^2+^ and MgPP_i_, for points corresponding to very low [Mg^2+^] and [MgPP_i_], the double reciprocal plots were nonlinear (Figure 2B,D). Most likely, under these conditions, free PP_i_ inhibited the reaction and, as [Mg^2+^] increased, the ratio of [MgPP_i_]/[PPi_free_] also increased, which resulted in relieving the inhibition (the linear portions of the double-reciprocal plots). This was similar to what was observed in Figure 1, where excess of total PP_i_ inhibited the reaction, whereas excess of MgCl_2_ increased the activity. The apparent *K*_m_ values for Mg^2+^ and MgPP_i_, calculated from the linear portion of the double-reciprocal plots were 0.13 and 0.06 mM, respectively (Figure 2B,D).

Earlier kinetic studies on barley UGPase yielded the *K*_m_ value with total PP_i_ of 0.04 mM [20]. This should be compared with *K*_m_ of 0.06 mM with MgPPi, as obtained in the present study. A possible explanation for this discrepancy is that in the earlier study the concentration of total magnesium was fixed at 5 mM, and it was total PP_i_ that was varied from 0.11 to 20 mM. Under these conditions, up to the total [PP_i_] of ca. 1 mM, most of PP_i_ was complexed as MgPPi and, importantly, free PPi was low or very low. At total [PP_i_] above 2 mM, a notable substrate inhibition was observed [20], which can be now explained by the effect of free PP_i_ rather than MgPP_i_. In contrast to these earlier results, the current setup of kinetic assays was different (Figure 2). Instead of fixed total [Mg] and varying total [PP_i_], it was the concentration of total PP_i_ that was fixed (at 0.5 mM), whereas [Mg^2+^] was varied. Thus, at very low [Mg^2+^], the assays contained huge excess of free PP_i_ over MgPP_i_, with the former inhibiting the reaction. This most likely accounts for a non-linear kinetics observed on double-reciprocal plots at low [Mg^2+^] and low [MgPP_i_] (Figure 2B,D).

Overall, the data presented in Figure 1 and Figure 2 strongly suggest that MgPP_i_ rather than total PP_i_ is true substrate of the reverse reaction of barley UGPase. This role of Mg^2+^ is not surprising, since it is absolutely required also for the forward reaction, with MgUTP (but not free UTP), serving as substrate [10,25]. For the reverse reaction, Mg^2+^ can perhaps on its own also contribute to the activity, as its assay concentration changes in step with changes of [MgPP_i_] (Figure 2). In this case, however, Mg^2+^ may be considered a cofactor rather than a substrate, since it does not undergo any change during the reaction. It should be mentioned here that other substrates of UGPase (Glc-1-P and UDPG in the forward and reverse reaction, respectively) are rather unlikely to act as Mg-bound species. Both of these compounds do bind Mg, albeit weakly, and their stability constants are low (e.g., *K* value of 0.06 mM^−1^ for Mg-bound Glc-1-P [23]). Binding of Mg^2+^ to UDPG was reported to be of similar strength to that of Mg^2+^ binding to Glc-1-P [15]. Nevertheless, by analyzing crystal structure of UGPase from *Helicobacter pylori*, it has been observed that Mg^2+^ binds to both UTP and UDPG [25]. This is consistent with an ordered bi-bi sequential mechanism, which is characteristic for both prokaryotic and eukaryotic UGPases [10,25,26] and, generally, other types of pyrophosphorylases [27]. One possibility is that free UDPG may interact with an already UGPase-bound Mg^2+^, which would then act as a cofactor.

To our knowledge, this is the first report where studies on pyrophosphorolysis reaction of UGPase were carried out with defined concentrations of MgPP_i_. MgPP_i_ was previously proposed as the true substrate for UGPases from *Sorghum* [28] and potato tubers [8], although without evidence arising from MgPP_i_ kinetics. It seems likely that, besides UGPase, MgPP_i_ is also a true substrate for other structurally-related pyrophosphorylases, e.g., UDP-sugar producing pyrophosphorylases [29] and, perhaps, for ADP-glucose pyrophosphorylase, a key enzyme of starch synthesis. All these enzymes require Mg^2+^ for activity and use PP_i_ and nucleoside-diphosphate sugar as substrates of their reverse reaction [2,9,27,29,30,31].

PP_i_-dependent reactions are frequently more active, when cytosolic [Mg^2+^] increases and when energy supply in the form of nucleoside triphosphates (e.g., ATP, UTP) is limited, as in anoxia/hypoxia [3,4,32]. An excess of [Mg^2+^] over total [PP_i_] appears to be a key requirement for the involvement of MgPPi, rather than free PP_i_, as substrate not only in the case of UGPase, but also for PP_i_-dependent phosphofructokinase [33], the latter being actually inhibited by free PP_i_ [34]. PP_i_ is also an inhibitor of UGPase forward reaction, as found by in vivo studies on plants with knocked out H^+^-pumping vacuolar pyrophosphatase [35]. MgPP_i_ complexes are true substrates for both H^+^-pumping and non-proton-pumping pyrophosphatases [22,36]. Interactions between Mg^2+^ and PP_i_/nucleotides and their role as substrates and regulators of cellular metabolism have been discussed in more detail in our recent works [4,7,14].

### 2.2. Effects of Selected PP_i_ and P_i_ Analogs on UGPase Activity 

**Figure 3:**
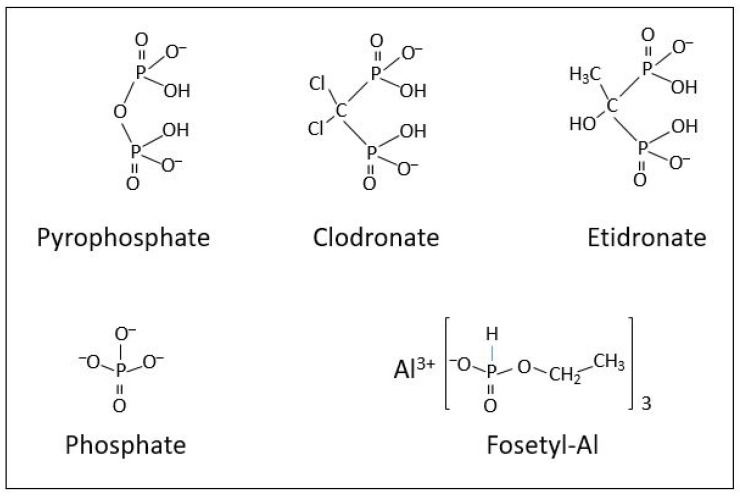
Structures of inorganic pyrophosphate and inorganic phosphate, along with their phosphonate analogs that were used in this study.

In an attempt to extend the studies on PP_i_ and Mg^2+^ interactions for UGPase activity, we tested the effects of phosphonates, compounds in which the active group is phosphite ion (HPO_3_^2−^) rather than phosphate (PO_4_^3−^) as in PP_i_ (Figure 3). Phosphite contains one less oxygen than phosphate, and is more soluble than phosphate, thus making its uptake by plant tissues more efficient. Whereas clodronate and etidronate are PP_i_ analogs and belong to bisphosphonates, fosetyl-Al is an analog of phosphate rather than of PP_i_ and is a representative of monophosphonates (Figure 3). Phosphonates, in general, have been demonstrated to greatly increase sizes of the PP_i_ pool in several species of pathogenic *Phytophthora palmivora*, and a phosphonate treatment of *Phytophthora citrophthora* led to a ten-fold increase in UGPase activity, possibly to compensate for decreases in the UDPG pool [37]. It has also been shown that addition of phosphonate causes inhibition of a pyrophosphatase in *Phytophthora palmivora* and *Saccharomyces cerevisae* [37].

**Figure 4:**
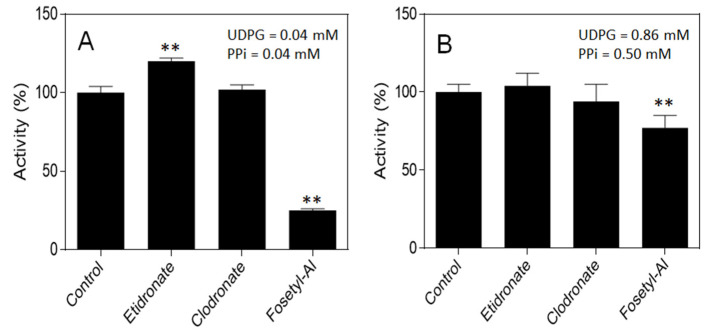
Effects of etidronate, clodronate and fosetyl-Al on activity of barley UGPase, assayed for the pyrophosphorolysis direction, at low (**A**) and saturating (**B**) concentrations of both UDPG and PP_i_. The phosphonates were at 1 mM each. Statistical significance in relation to control: ** (*p* < 0.01).

The possible effects on UGPase activity of fosetyl-Al, clodronate and etidronate were measured using standard assay conditions in the pyrophosphorolysis direction, with the exception that the substrates were kept either at close to their *K*_m_ values, i.e., 0.03 mM and 0.04 mM for UDPG and PP_i_, respectively [20], or at their saturating concentrations upon addition of 1 mM of the respective inhibitors (Figure 4). Of the three compounds, only fosetyl-Al had a considerable effect on UGPase activity, acting as inhibitor, especially under non-saturating substrate conditions (Figure 4A). The fact that fosetyl-Al was a stronger inhibitor at substrate non-saturating conditions suggested that it binds close to or at the binding site for one of the substrates of UGPase. 

**Figure 5:**
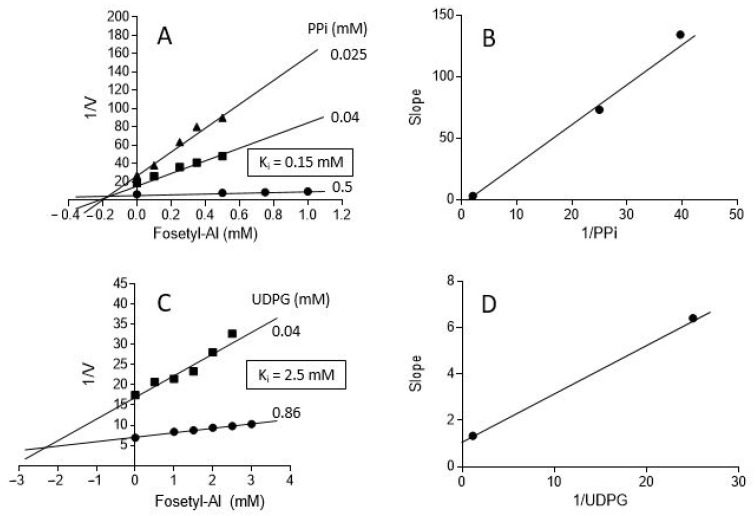
Fosetyl-Al inhibition of barley UGPase pyrophosphorolytic activity at varied PP_i_ or UDPG. (**A**) UGPase activity (V) was assayed with varied concentrations of fosetyl-Al, at three different PP_i_ concentrations and with UDPG fixed at 0.86 mM. (**B**) Slopes of the lines from panel (**A**) versus 1/[PP_i_]. (**C**) UGPase activity (V) was assayed with varied concentrations of fosetyl-Al, at two different concentrations of UDPG and with PP_i_ fixed at 0.5 mM. (**D**) Slopes of the lines from panel (**C**) versus 1/[UDPG]. Please see the text for rationale for (**B**,**D**) panels.

We examined the effects of fosetyl-Al in more detail using Dixon plots [24,38], where activity of the enzyme, assayed in the pyrophosphorolysis direction, was determined at various concentrations of fosetyl-Al and using a fixed saturating concentration of one substrate and different concentrations of the second reactant (Figure 5). Inhibition constants (*K*_i_) for fosetyl-Al against each of the UGPase substrates could be estimated from intersection of lines on the Dixon plot [24,38]. The *K*_i_ values estimated from Dixon plots were approximately 0.15 mM (with PP_i_ varied) and 2.3 mM (with UDPG varied). The intersection points above the *X*-axis have indicated that the inhibitor is not uncompetitive or noncompetitive versus PP_i_ or UDPG. Plotting of the slopes versus the inverse of the substrate concentration allowed us to determine whether the inhibition was of pure competitive or mixed character; in the case of competitive inhibition the crossing trough “zero” would indicate that addition of infinite amounts of substrates prevents inhibitor effects. In conclusion, based on data in Figure 5, the nature of the fosetyl-Al inhibition versus PP_i_ is near-competitive, while its inhibition versus UDPG appears to be of mixed character [24,38].

The fact that fosetyl-Al, but not clodronate nor etidronate, served as a relatively strong UGPase inhibitor (Figure 4) is surprising. Fosetyl-Al is not a PP_i_ analog (Figure 3) and nonetheless it appears to bind near or at the PP_i_ binding site rather than interfering with the UDPG-binding site, as suggested by its lower *K*_i_ versus PP_i_ (Figure 5A) compared to its *K*_i_ versus UDPG (Figure 5C). The stability constant for fosetyl-Al and Mg^2+^ is unknown, but it is probably similar, or lower, than that of phosphate, its close analog (Figure 3). Phosphate itself was earlier found to inhibit UGPase activity, but its effect was weak [39]. Phosphate is also a weak chelator with magnesium, with the stability constant for MgP_i_ complex of 0.5 mM^−1^ [23]; thus, fosetyl-Al is rather unlikely to act as a complex with Mg^2+^ during its inhibition of the UGPase. It should be also emphasized that assays in Figure 4 and Figure 5 contained 5 mM MgCl_2_, and that the highest total [PP_i_] was 0.5 mM. This assured that more than 80% of total PP_i_ was in the form of MgPP_i_, a true substrate of the reaction.

Fosetyl-Al was previously used within the agricultural sector as a treatment against *Phytophthora* infections [40] and it may act either directly on the pathogen or indirectly by stimulating host-defense responses [18,41]. Fosetyl-Al has been shown to be easily assimilated and to be translocated throughout the plant through the phloem. In plants treated with fosetyl-Al, several changes in physiology have been observed, such as decreased fertility and obstructed pollen tube growth [40]. However, not much is known about specific targets for fosetyl-Al [42] and there were no studies on effects of this compound on UGPase activity. UGPase produces UDPG, which is a substrate for hundreds of different glycosylation reactions involving, e.g., glycosylation of proteins, polysaccharides and lipids, among other compounds. Protein glycosylation is one of the mechanisms employed in host-pathogen interaction, affecting host resistance and/or pathogen virulence [43,44]. UDPG is also a precursor to many sugars, including those that may inhibit growth of *Phytophtora* [45]. However, whether the activity of UGPase, in either a plant or a pathogen or both, is directly affected by fosetyl-Al application is unknown at present.

More studies on effects of phosphonates on PP_i_-utilizing reactions are required. UGPase belongs to a family of enzymes sharing a common structural blueprint [26,46], generally named UDP-sugar-producing pyrophosphorylases (USPP). All these enzymes catalyze fully reversible reactions, using UTP and sugar-1-P to produce UDP-sugar and PP_i_ [2]. These enzymes frequently have overlapping specificities for sugar-1-P and UDP-sugar as substrates/products [29,31,46], and thus it is difficult to distinguish between them when assayed in crude cellular extracts. For instance, all USPPs can produce/utilize UDPG as either a specific (UGPase) or non-specific (other USPP enzymes) product/substrate [2]. Despite an important role played by UDP-sugars in many cellular processes, no specific inhibitors have been described for any of the USPPs [47,48]. Once such inhibitors are identified, the extent of sensitivity to inhibitors may represent a distinctive feature of a given USPP enzyme when studied in crude extracts of any plant species [2,47,48]. Thus, phosphonates, including fosetyl-Al, are feasible candidate compounds to screen against each of the USPP activities.

## 3. Material and Methods

### 3.1. Materials

Purified recombinant (*E. coli*-expressed) UGPase from barley (*Hordeum vulgare*) [20,49] was used for assays. Before use, the enzyme was diluted up to 1:250 fold in a buffer containing 100 mM Hepes-NaOH (pH 7.5), 5 mM MgCl_2_ and 15% (*w*/*v*) sucrose. Fosetyl-Al was from ChemService Inc. (West Chester, PA, USA), whereas clodronate and etidronate were from Sigma (D4434 and P5248, respectively)

### 3.2. UGPase Assays

The UGPase was assayed in its reverse reaction (pyrophosphorolysis). During assays, the formation of Glu-1-P was coupled to the activity of phosphoglucomutase (PGM) (Sigma P3397) and to the activity of glucose-6-phosphate dehydrogenase (G6PDH) (Roche #10127671001). Standard reaction mixture (in 1 mL) contained 100 mM Hepes-NaOH (pH 7.5), 5 mM MgCl_2_, 5–20 µL of UGPase, 2 units of each of PGM and G6PDH, 0.3 mM NADP and 0.5 mM PP_i_. Reactions were initiated with 0.86 mM UDPG, and the formation of NADP was monitored spectrophotometrically (Beckman DU 530) at 340 nm. One unit of UGPase activity was defined as the formation of 1 µmol of NADPH per min [20].

### 3.3. Magnesium and PP_i_ Requirements

For the effects of total concentrations of magnesium and PPi on UGPase activity (see Figure 1), EDTA was added to eliminate Mg from the assays. For kinetic analyses (see Figure 2), assays contained 0.5 mM UDPG, 0.5 mM PP_i_ and varied concentrations of MgCl_2_ (from 0.07 to 5 mM). Reactions were started with MgCl_2_ and were run against control assays with no MgCl_2_. Other reactants were as in standard assay. Concentrations of Mg^2+^ and MgPP_i_ were calculated for each assay, using the stability constant for MgPP_i_ of 1.2 mM^−1^ [23].

### 3.4. Effects of Phosphonates on UGPase Activity

To evaluate the effects of phosphonates on UGPase activity (see Figure 4), the assays were carried out at low and saturating concentrations of both UDPG and PP_i_. At low concentration conditions, both UDPG and PP_i_ were at 0.04 mM, whereas at high concentration conditions, UDPG and PPi were at 0.86 mM and 0.5 mM, respectively. The phosphonates were at 1 mM each. Other reactants were as in standard assay (see above).

In order to assess in more detail (via Dixon plots) the inhibition of UGPase by fosetyl-Al and to determine apparent inhibition constants (*K*_i_) for this compound, two series of experiments were carried out (see Figure 5): one with varying [fosetyl-Al] at three varied total PP_i_ concentrations (0.025 mM, 0.04 mM and 0.5 mM) and UDPG at constant saturating concentration (0.86 mM), and a second with varying [fosetyl-Al] at two varied UDPG concentrations (0.04 mM and 0.86 mM) and PP_i_ at constant saturating concentration (0.5 mM). The rationale behind the design of those experiments, and for estimation of the *K*_i_s, was from Segel [24]. Other than that, standard assay conditions for pyrophosphorolysis reaction were maintained (see above).

### 3.5. Statistical Analyses

All assays were done in 2 to 5 repeats for each experimental point. Student *t* test calculations were performed using GraphPad Prism 6 statistics software.(GraphPad Software, La Jolla, CA, USA).

## Data Availability

Not applicable.
